# Intracranial Lesions in Children and Adolescents with Morbid Obesity

**DOI:** 10.4274/balkanmedj.2015.1541

**Published:** 2017-03-28

**Authors:** Ayça Törel Ergür, Sevinç Odabaşı Güneş, Sinan Tan, Ü. Ayşe Tandırcıoğlu

**Affiliations:** 1 Department of Pediatrics, Division of Pediatric Endocrinology, Kırıkkale University Faculty of Medicine, Kırıkkale, Turkey; 2 Department of Radiology, Kırıkkkale University Faculty of Medicine, Kırıkkale, Turkey; 3 Department of Pediatrics, Kırıkkale University Faculty of Medicine, Kırıkkale, Turkey

**Keywords:** Intracranial lesions, children, morbid obesity, magnetic resonance imaging

## Abstract

**Background:**

Intracranial lesions may affect the hypothalamo-hypophyseal axis and lead to some neuro-endocrinological dysfunctions (hyperphagia, sleep disorders and hormonal dysfunctions). There is a very limited number of studies about childhood obesity and intracranial lesions.

**Aims:**

To evaluate the incidence of intracranial lesions and its role in clinical symptoms and aetiology in cases with morbid obesity who have been admitted to the paediatric endocrinology department with this complaint.

**Study Design:**

Cross-sectional study.

**Methods:**

A total of 120 cases admitted to the paediatric endocrinology department with the complaint of morbid obesity between 2002 and 2015 were included in this study. A detailed history was taken and a physical examination was performed; biochemical, hormonal parameters were evaluated. Contrast dynamic magnetic resonance imaging was performed in order to visualize cranial pathologies.

**Results:**

An intracranial lesions was detected in 16.6% of the patients and 55% of these lesions were adenoma of the hypophysis. Prolactin levels were increased in six patients but front hypophyseal hormone levels were within normal range in the rest of the patients. Growth velocity of the patients was not affected.

**Conclusion:**

In our study, the incidence of intracranial lesions in children and adolescents with morbid obesity was much higher than in the normal population. According to this data, we are of the opinion that contrast dynamic magnetic resonance imaging is helpful in children with morbid obesity for the early detection of the mass before it causes any clinical or neurological symptoms and in the prevention of future complications.

Many neuro-endocrinological factors, including intracranial lesions (ICLs), play a role in the aetiology of childhood obesity, which is a very common disease in our century. Lesions (inflammatory, traumatic, tumours, etc.) in the ventromedial hypothalamus, paraventricular hypothalamus, lateral hypothalamus or arcuate nucleus may lead to hyperphagia, hormonal deficiencies, low basal metabolism rate, decrement in sleep and cause obesity. In particular, these kind of ICLs may affect the hypothalamo-hypophyseal axis, depending on the localization of the lesion, and may cause growth hormone (GH) and other hypophyseal hormonal deficiencies (1,2). Tumours of the hypophysis are not very common in childhood and adolescents, and incidence was reported to be 1/1000000 (3). Most of these tumours are not malignant but they cause symptoms by increasing hormonal secretion or by the exerting of a mass effect on the brain. In another study of 225 asymptomatic healthy children, the percentage of cranial abnormalities besides sinusitis found in magnetic resonance imaging (MRI) of the brain was reported to be 8.8% (4).

Although ICL are important in the aetiology of obesity, there is not any investigation about the incidence of ICL in cases with morbid obesity (MO). The purpose of this study is to evaluate the incidence of ICL and its role in clinical symptoms and the aetiology in cases with MO.

## MATERIAL AND METHODS

One hundred twenty patients aged between 3 and 18 years who were admitted to the Paediatric Endocrinology Department with a complaint of obesity were involved in the study. A detailed history of patients was taken, along with anthropometric measurements [height, weight, body mass index (BMI) and percentiles, pubertal Tanner staging, bone age], and a physical examination was made. Biochemical, hormonal parameters [fasting blood glucose and insulin level (FBG, Fin), lipid parameters, thyroid function tests, diurnal cortisol and oral glucose tolerance test (OGTT)] were evaluated. Growth velocities of patients were calculated. MO was defined as BMI upper than 99. percentile (SD scores: 2.33) according to sex and age (5). Homeostasis model assessment-insulin resistance (HOMA-IR) was defined as [FBG (mg/dL) x fasting blood insulin (IU/L) / 405]. Cut-off levels were defined as 2.22 in pre-pubertal girls, 2.67 in pre-pubertal boys, 3.82 in pubertal girls and 5.22 in pubertal boys (6). Impaired fasting glucose was diagnosed as 100-125 mg/dL and glucose intolerance was diagnosed as 140-199 ng/mL during OGTT (7). Blood cortisol levels were obtained at 8:00 a.m. and at midnight during sleep. A morning cortisol level above 20 µg/dL and a midnight cortisol level above 1.8 µg/dL were determined as pathologic (8). Contrast dynamic MRI (CDMRI) (Philips Achieva 1.5-T scanner, Best, the Netherlands) was obtained in order to evaluate hypothalamus and hypophysis. MRI protocol of the hypophysis and sella region was performed in the unenhanced T1- and T2- weighed images in coronal and sagittal planes and post-contrast dynamic T1- weighed coronal and sagittal images were taken after intravenous injection of gadolinium at the standard dose. MRI examination of hypophysis and sella region were evaluated for the height and anatomic details of the hypophysis, enhancement pattern of the lesion and extension into the suprasellar region. The height of hypophysis was evaluated according to age and sex. Height >+2 SD was defined as hypophyseal hyperplasia (HH), height below <-2 SD was defined as hypophyseal hypoplasia (9). Adenohypophyseal hormones (luteinizing hormone, follicle stimulating hormone, adrenocorticotrophic hormone, thyroid-stimulating hormone, prolactin, GH were obtained from the cases with ICL lesions and chemiluminescence method was used in evaluation (10). Early morning prolactin levels above 20 ng/mL in at least two different measurements were determined as pathologic. Written informed consent was obtained from all the patients and their parents. The local ethics committee approved this study.

### Statistical analysis

A commercially available statistical software package (SPSS 21.0 for Windows, Chicago, III. USA) was employed for all statistical analyses. Distributions of the values were analysed with the Kolmogorov-Smirnov test and values were presented as mean. Comparison of anthropometric measurements, plasma glucose, insulin levels and HOMA-IR levels of the patients with ICL lesions and patients without lesion were made using Student’s t-test. A p-value of less than 0.05 was considered statistically significant.

## RESULTS

Anthropometric, biochemical and hormonal parameters of all the cases are given in Table 1. In total, 20 cases (16.6%) had an ICL. Characteristics of these 20 cases are given in Table 2.

**Figure 1:**
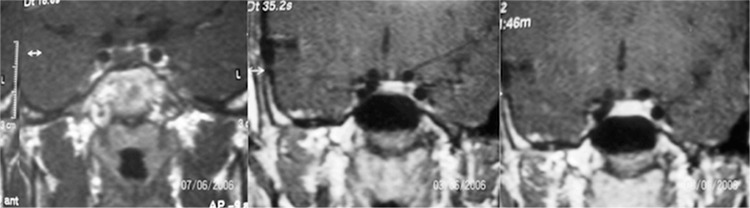
12x7 mm bilobulated hypointense lesion (hypophyseal adenoma), which is seen in 35. second in contrasted dynamic MRI, but not in 3 minute.

**Figure 2:**
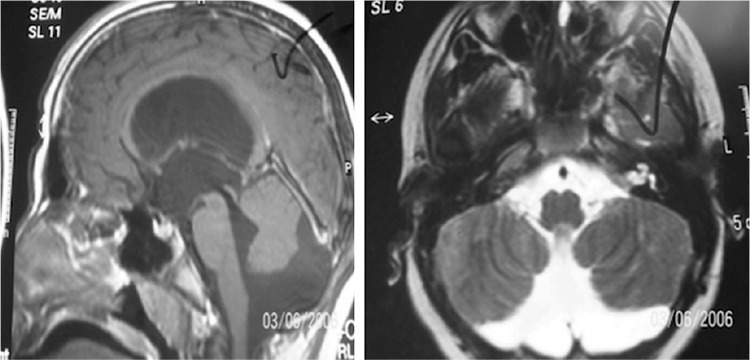
Dandy-Walker Syndrome which is characterized by agenesis or hypoplasia of the cerebellar vermis, cystic dilatation of the fourth ventricle, and enlargement of the posterior fossa.

Most of the cases had adenoma of the hypophysis and cases with adenoma of hypophysis constituted 55% of the cases with ICL (Figure 1). Two patients had HH and one of these patients also had a 4.4x4 mm microadenoma. Other ICLs were Dandy-Walker Syndrome (Figure 2) arachnoid cyst, rathke cleft cyst, and pineal cyst. Six of the patients with ICL had high prolactin levels according to the age and sex (case number 11,12,13,15,18,20). None of the patients had hormonal abnormalities except these six cases. One of these cases with high prolactin levels (>20 ng/mL) had headache, menstrual irregularity and galactorrhea, the other one had only headache. History of the cases was negative for neurological or endocrinological symptoms (polyuria, polydipsia, blurred vision, vomiting, precocious puberty). Five of the cases had glucose intolerance and one of the cases had impaired fasting blood glucose. Growth velocity (GV) of the cases was within normal range. Cases with ICL lesions (group 1) and cases without ICL (group 2) were compared. There was no statistically significant difference between group 1 and group 2 (p>0.05).

## DISCUSSION

IC lesions show mass effect to the surrounding tissue, resulting in neurologic/neuro-ophthalmologic symptoms or endocrinologic problems, including obesity (11). It has been accepted that besides causing hypothalamic injury lesions may impair signal transmission in the neuro-anatomic structure and cause obesity (2). However, in our study neuro-ophthalmic/neurologic examination or history was negative other than MO, which may make us think ICL. This situation suggests that this may delay the early diagnosis of the disease by paediatricians or paediatric endocrinologists or may lead to misdiagnosis.

In our study, the incidence of ICL lesions in MO patients is much more higher than that found in the literature. This finding indicates that ICLs are involved in the pathophysiology of the disease and intracranial imaging is important in the MO patient group.

Hypophyseal adenomas are very rare in childhood but they are the most common lesions of the hypophysis (12). Similarly, hypophyseal adenomas are found to be the most common lesions in our study, and the incidence within ICL lesions was 55%. This percentage is much higher than that found in the literature. This situation shows that a detailed physical examination and neurologic/neuro-ophthalmologic examination should be performed in MO patients.

Hyperinsulinism in patients with exogenous obesity leads to an increment in GV by increasing insulin-like growth factor 1 (IGF-1) production and bioavailability or showing a cross-reaction with IGF-1 receptors (1,13). By contrast, in patients with endocrinopathy, GV decreases and leads to short stature (1,14). However in our study, the GV of patients with ICLs was not affected, which may be misdiagnosed with exogenous obesity. None of the patients had a decrement in GV, demonstrating that weight gain increased without any clinical symptoms appearing. Taylor et al. (15) found similar results, which supports our study. A total of 176 patients with hypothalamo-pituitary lesions (craniaopharyngioma, optic glioma, arachnoid cyst, hamartoma, germ cell tumour) were evaluated retrospectively. It was concluded that 23% of the patients had obesity before diagnosis and 85% of these obese patients had normal GV. When development of symptoms was evaluated, it was recognized that BMI increment and weight gain appeared before other neurologic symptoms. In our study among patients with ICL lesions, there were only 2 (1%) cases who had symptoms besides weight gain, namely headache, and headache + menstrual irregularity + galactorrhea. According to this result, in cases with abnormal BMI or BMI increment with or without GV change, the history should be taken more carefully and a clinical evaluation should be done.

Our study involves a long period of 13 years, which makes our results more reliable. A limitation to this study is that there is not much study of the prevalence of ICL in the healthy, asymptomatic paediatric population. We consider our study also contributes a new perspective on this subject. The results show that, the incidence of ICL is more common in children and adolescents with MO than in thenormal population. Most of these lesions show no other endocrinological or neurological symptoms other than obesity. We would like to emphasize that after making a detailed physical and neurological examination, CDMRI in particular should be performed in cases with MO, so that the diagnosis of those cases without any other clinical symptoms could be made earlier and treatment offered.

## Figures and Tables

**Table 1:**
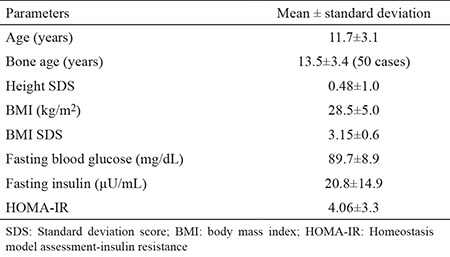
Anthropometric and biochemical parameters of the cases

**Table 2:**
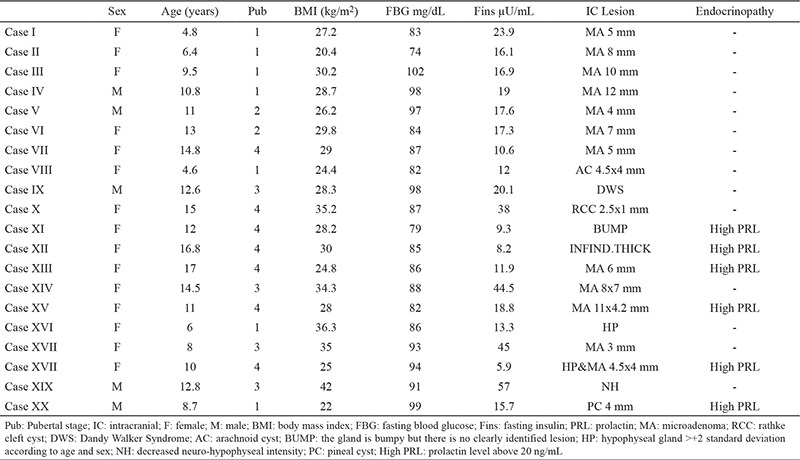
Anthropometric, hormonal and radiologic evaluation of cases with intracranial and intrahypophyseal lesions
